# LIF-activated Jak signaling determines Esrrb expression during late-stage reprogramming

**DOI:** 10.1242/bio.029264

**Published:** 2017-12-06

**Authors:** Delun Huang, Ling Wang, Jingyue Duan, Chang Huang, Xiuchun (Cindy) Tian, Ming Zhang, Young Tang

**Affiliations:** 1 State Key Laboratory for Conservation and Utilization of Subtropical Agro-Bioresources, Animal Reproduction Institute, Guangxi University, Nanning, Guangxi 530004, China; 2 Department of Animal Science, Institute for Systems Genomics, University of Connecticut, Storrs, CT 06269, USA

**Keywords:** Esrrb, Jak, LIF, Reprogramming, iPSC

## Abstract

The regulatory process of naïve-state induced pluripotent stem cell (iPSC) generation is not well understood. Leukemia inhibitory factor (LIF)-activated Janus kinase/signal transducer and activator of transcription 3 (Jak/Stat3) is the master regulator for naïve-state pluripotency achievement and maintenance. The estrogen-related receptor beta (*Esrrb*) serves as a naïve-state marker gene regulating self-renewal of embryonic stem cells (ESCs). However, the interconnection between *Esrrb* and LIF signaling for pluripotency establishment in reprogramming is unclear. We screened the marker genes critical for complete reprogramming during mouse iPSC generation, and identified genes including *Esrrb* that are responsive to LIF/Jak pathway signaling. Overexpression of *Esrrb* resumes the reprogramming halted by inhibition of Jak activity in partially reprogrammed cells (pre-iPSCs), and leads to the generation of pluripotent iPSCs. We further show that neither overexpression of *Nanog* nor stimulation of Wnt signaling, two upstream regulators of *Esrrb* in ESCs, stimulates the expression of *Esrrb* in reprogramming when LIF or Jak activity is blocked. Our study demonstrates that *Esrrb* is a specific reprogramming factor regulated downstream of the LIF/Jak signaling pathway. These results shed new light on the regulatory role of LIF pathway on complete pluripotency establishment during iPSC generation.

## Abstract (precis)

**Summary:** *Esrrb* is a downstream target and effector of LIF during reprogramming. Forced *Esrrb* expression accelerates pluripotency establishment in the absence of LIF signaling. The activation of *Esrrb* is LIF dependent in the reprogramming process.

## INTRODUCTION

Generation of induced pluripotent stem cells (iPSCs) (Takahashi and Yamanaka, 2006) leads to the establishment of pluripotency equivalent to embryonic stem cells (ESCs) without embryo destruction, by overexpressing the so-called Yamanaka factors, namely Oct4, Klf4, Sox2 and c-Myc (OKSM). However, to date, a complete understanding of pluripotency establishment has not been achieved. The cytokine leukemia inhibitory factor (LIF) activates the Janus kinas/signal transducer and activator of transcription 3 (Jak/Stat3) pathway, which serves as a key for the self-renewal of naïve-state pluripotent mouse ESCs (Matsuda et al., 1999; Nichols and Smith, 2009; Niwa et al., 1998; Smith et al., 1988; Williams et al., 1988). Stat3 activity also plays a fundamental role for naïve-state iPSC generation at late-reprogramming stage (Tang et al., 2012; van Oosten et al., 2012; Yang et al., 2010). A number of genes have been reported to be regulated by Stat3 and mediate LIF-independent mouse ESC self-renewal or iPSC reprogramming. These include *MnSOD*, *Klf4*, *Klf5*, *Nanog*, *Gbx2*, *Pim1*, *Pim3*, *Pramel7*, *Tfcp2l1*, *c-Myc* and *Foxm1* (Aksoy et al., 2007; Cartwright et al., 2005; Casanova et al., 2011; Festuccia et al., 2012; Hall et al., 2009; Martello et al., 2013; Niwa et al., 2009; Parisi et al., 2008; Sheshadri et al., 2015; Tai and Ying, 2013; Tan et al., 2014; Ye et al., 2013). We also found that Jak/Stat3 regulates key epigenetic change during the reprogramming process (Tang et al., 2012). However, a question remains as how exactly Jak/Stat3 activity regulates pluripotency establishment during the reprogramming process. A better understanding of the Stat3-regulated downstream targets/effectors is necessary, and will further facilitate the naïve-state iPSC generation across different species including humans (De Los Angeles et al., 2012).

The nuclear receptor estrogen-related receptor beta (*Esrrb*) is a canonical Wnt pathway effector negatively regulated by glycogen synthase kinase 3 (GSK3)/T-cell factor 3 (Tcf3) in naïve-state ESCs, and its overexpression can sustain ESC self-renewal that mimics the inhibition of GSK3 (Martello et al., 2012). In ESCs, the expression of *Esrrb* can also be regulated by *Nanog*, and overexpressing *Esrrb* promotes complete reprogramming from Nanog-null partially reprogrammed iPSCs (pre-iPSCs), and can sustain LIF-independent ESC self-renewal similarly to *Nanog* (Festuccia et al., 2012). *Nanog* is not a GSK3 downstream effector (Martello et al., 2012; Silva et al., 2009). This indicates that *Esrrb* is subjected to multi-upstream signaling regulation for pluripotency establishment and maintenance. However, whether *Esrrb* is regulated under LIF-mediated Jak/Stat3 signaling during the reprogramming process is not clear. In this study, we screened the expression of key pluripotency genes regulated by Jak/Stat3 and LIF activities in reprogramming. We describe the identification of *Esrrb* as an important effector downstream of LIF/Jak/Stat3 signaling for completely reprogrammed iPSC generation, with its expression dependent on LIF pathway activation.

## RESULTS

### Esrrb is activated by LIF/Jak signaling during the reprogramming process

**Fig. 1.**
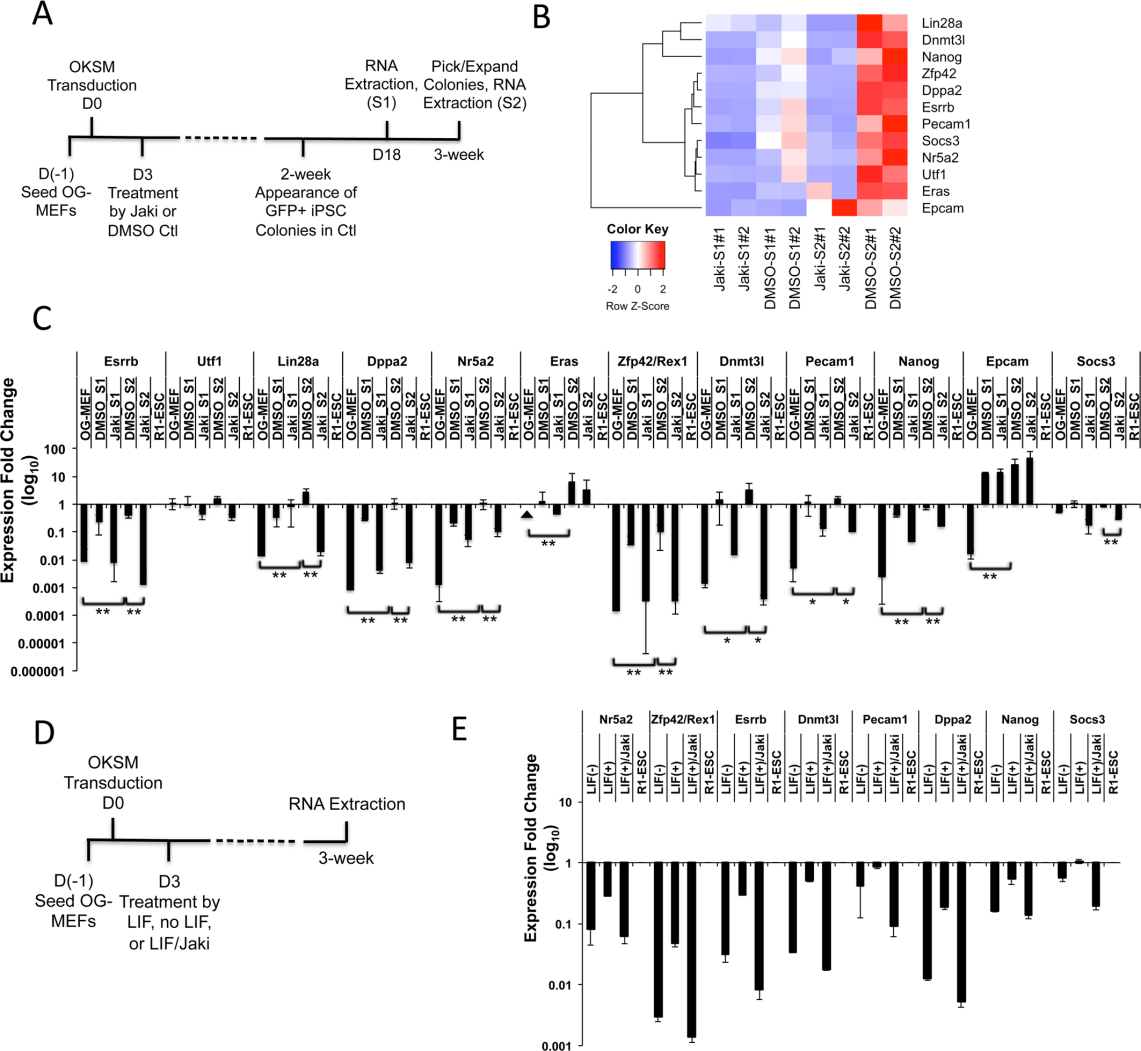
**Esrrb is regulated by LIF and Jak/Stat3 activity in reprogramming.** (A) Schematic diagram depicting the reprogramming process and dates (S1 and S2) for RNA-seq sample collection from reprogrammed cells. (B) Heatmap of FPKM value comparison for key pluripotent genes plus the Stat3 activity indicator Socs3 under Jaki or DMSO treatment at reprogramming stage S1 and S2. The relative abundance is indicated by color (blue, lower abundance; red, higher abundance). (C) qRT-PCR analysis of pluripotent genes in reprogrammed cells collected at Jaki or DMSO treatment at reprogramming stage S1 and S2. Values are relative to R1-ESC standard. Data are mean±s.d. from three independent biological repeats. The arrowhead indicates that expression was not detected. *P<0.05, **P<0.01. (D) Schematic diagram depicting the reprogramming process and dates for the LIF-deprivation MEF reprogramming experiment. (E) qRT-PCR analysis of pluripotent genes in reprogrammed cells with or without LIF, or with LIF plus Jaki treatment at a 3-week time point. Values are relative to R1-ESC standard. Data are mean±s.d. from two independent biological repeats.

Previous studies of reprogramming dynamics towards naïve-state pluripotency have identified a number of pluripotent genes for which expression in reprogramming stringently marks the development to pluripotent iPSC state (Buganim et al., 2012; Polo et al., 2012). These genes include *Esrrb*, *Utf1*, *Lin28a*, *Dppa2*, *Nr5a2*, *Eras*, *Rex1/Zfp42*, *Dnmt3l*, *Pecam1*, *Nanog* and *Epcam* (Buganim et al., 2012; Polo et al., 2012). To understand the expression of these genes relevant to Jak/Stat3 activity in reprogramming, we utilized the RNA-seq data recently generated by us (GEO accession number GSE97261) (Wang et al., 2017b), where we blocked Jak/Stat3 activity using a well-studied Jak-specific inhibitor (Jak inhibitor I, Jaki) (Niwa et al., 2009; Thompson et al., 2002) during the reprogramming of mouse embryonic fibroblasts (MEFs) to iPSCs (Fig. 1A). These MEFs have green fluorescent protein (GFP) expression controlled by the Oct4 distal enhancer region (OG-MEFs), and total RNAs of reprogrammed OG-MEFs were analyzed on reprogramming day 18 (Stage 1, S1) and 3 weeks (S2) after retroviral OKSM infection (Fig. 1A). Heatmap analysis of the RNA-seq data reveals that the majority of the eleven pluripotency-predicting genes are downregulated by Jaki inhibition after 3 weeks of reprogramming (Fig. 1B). Quantitative reverse-transcription polymerase chain reaction (qRT-PCR) analysis further confirmed that except for three genes (*Utf1*, *Eras* and *Epcam*), all other pluripotent markers including *Esrrb* are significantly upregulated after 3 weeks of reprogramming in DMSO control, but inhibited when Jak/Stat3 activity is blocked (Fig. 1C).

We previously showed that similar to the Jaki treatment, deprivation of LIF cytokine (no LIF and feeder-cell free) during OG-MEF reprogramming resulted in the generation of only GFP-negative colonies (Tang et al., 2012). If the stimulation of these pluripotency-predicting genes in reprogramming is specifically controlled by Jak/Stat3 activity, we shall observe similar results when LIF cytokine, the stimulator of Jak/Stat3 signaling is depleted during mouse iPSC induction. We then compared the expression of these genes in MEFs reprogrammed by retroviral OKSM transduction and with or without LIF cytokine at a 3-week time point (Fig. 1D,E). Indeed, we found that the expression of these genes is significantly inhibited by depletion of LIF cytokine, to levels comparable to the Jaki treatment (Fig. 1E). Similar results were also observed when LIF is blocked using a LIF-neutralizing antibody (LIFAb) in reprogramming (Figs S1 and S2). Thus, the LIF-regulated Jak activity specifically stimulates pluripotency marker genes including Esrrb that are tightly associated with pluripotency development during the somatic cell reprogramming process.

### Esrrb promotes complete reprogramming in the presence of Jak/Stat3 inhibition

**Fig. 2.**
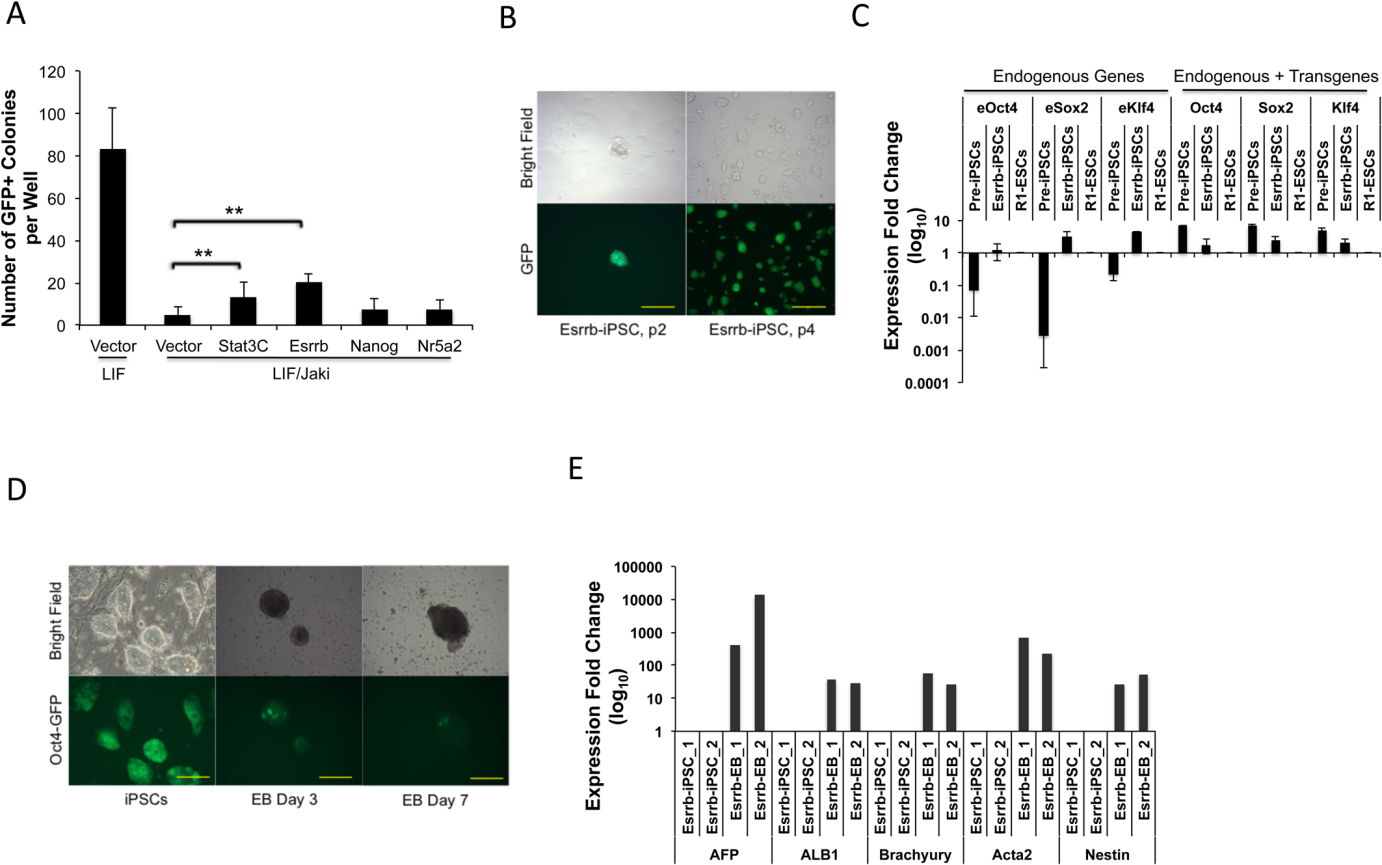
**Esrrb promotes complete reprogramming blocked by Jak/Stat3 inhibition.** (A) Pre-iPSCs were expanded and seeded into 24-well plates, infected with vector control or virally expressed Stat3C, Esrrb, Nanog or Nr5a2, and cultured in the presence of LIF or LIF plus Jaki. GFP+ colonies were counted 2 weeks after viral infection. Data are mean±s.d. from four independent experiments. **P<0.01. (B) The Esrrb induced putative iPSC colonies cultured in 2i/LIF medium at different passages (p). Scale bars: 120 μm (p2) and 625 μm (p4). (C) qRT-PCR analysis of endogenous (eOct4, eSox2, eKlf4) and total (endogenous plus viral transgene) expression of reprogramming factors in Esrrb-induced putative iPSC colonies at passage 3, together with the parental pre-iPSCs remaining in Jaki treatment. R1-ESC was used as the control. Data are mean±s.d. derived from two different cell lines. (D) EB formation from original Esrrb-induced iPSCs at days 3 and 7 of differentiation, with gradually silenced Oct4-GFP expression. Scale bars: 250 μm. (E) qRT-PCR analysis for relative expression levels of the three germ layer markers at day 14 of EB differentiation (endoderm: AFP, ALB1; mesoderm: Brachyury, Acta2; ectoderm: Nestin). The gene expression values of two differentiated EB lines were relative to their parental iPSCs.

We wanted to evaluate the functional significance of these pluripotent genes regulated by Jak/Stat3 activity for complete reprogramming. We utilized the pre-iPSCs isolated at a 3-week reprogramming point (Fig. 1A), which remained GFP-negative and continuous OKSM transgene expression under Jaki treatment (Tang et al., 2012). Overexpressing a constitutively active form of *Stat3* (Stat3C) (Bromberg et al., 1999) in these pre-iPSCs led to significantly increased GFP-positive (GFP+) colonies within 2 weeks in the presence of Jaki, further confirming a specific blocking of Stat3 signaling by Jaki treatment in halted reprogramming (Fig. 2A). We tested three candidate genes (*Esrrb*, *Nanog* and *Nr5a2*) for their overexpression on reprogramming of the pre-iPSCs. We chose these genes because *Nanog* was shown to upregulate *Esrrb* in ESCs (Festuccia et al., 2012), and *Nr5a2* was reported as a Wnt-regulated transcription factor that can stimulate the expression of *Oct4*, *Nanog* and *Tbx3* in ESCs (Wagner et al., 2010), and can replace *Oct4* for iPSC induction (Heng et al., 2010). Out of multiple trials, we consistently observed a significant increase of GFP+ colonies in 2-3 weeks by overexpression of Esrrb (to ∼25% of the GFP+ colonies developed in the absence of Jaki), whereas overexpressing *Nanog* or *Nr5a2* had negligible effects (Fig. 2A). Similar results were obtained when we overexpressed *Esrrb*, *Nanog*, *Nr5a2* and three other genes (*Klf2*, *Lin28*, and *Prdm14*) using two additional lines of pre-iPSCs (Fig. S3).

The GFP+ colonies induced by *Esrrb* overexpression can be further expanded in 2i/LIF, the restrictive medium for ground state pluripotency (Silva et al., 2009; Ying et al., 2008) (Fig. 2B). In contrast to their parental pre-iPSCs, the *Esrrb*-induced iPSCs showed expression of endogenous pluripotent genes at levels comparable to ESCs, including *Oct4*, *Sox2*, *Klf4*, *Nanog*, *Rex1*, *Dppa3* and *Nr5a2*, and silenced the transgene expression (Fig. 2C; Fig. S4). Furthermore, upon removal of the LIF cytokine, these *Esrrb*-induced iPSCs readily formed embryoid bodies (EBs), with gradual silencing of the Oct4-GFP fluorescence (Fig. 2D), and demonstrated three-germ layer differentiation (Fig. 2E; Fig. S5). These iPSCs also showed the ability of differentiation into beating cardiomyocytes (Movie 1). Taken together, our data demonstrate that during somatic cell reprogramming, the activation of *Esrrb* is one of the key effectors downstream of Jak/Stat3 for the complete pluripotency establishment.

### The expression of Esrrb depends on LIF/Jak pathway activity in reprogramming

**Fig. 3.**
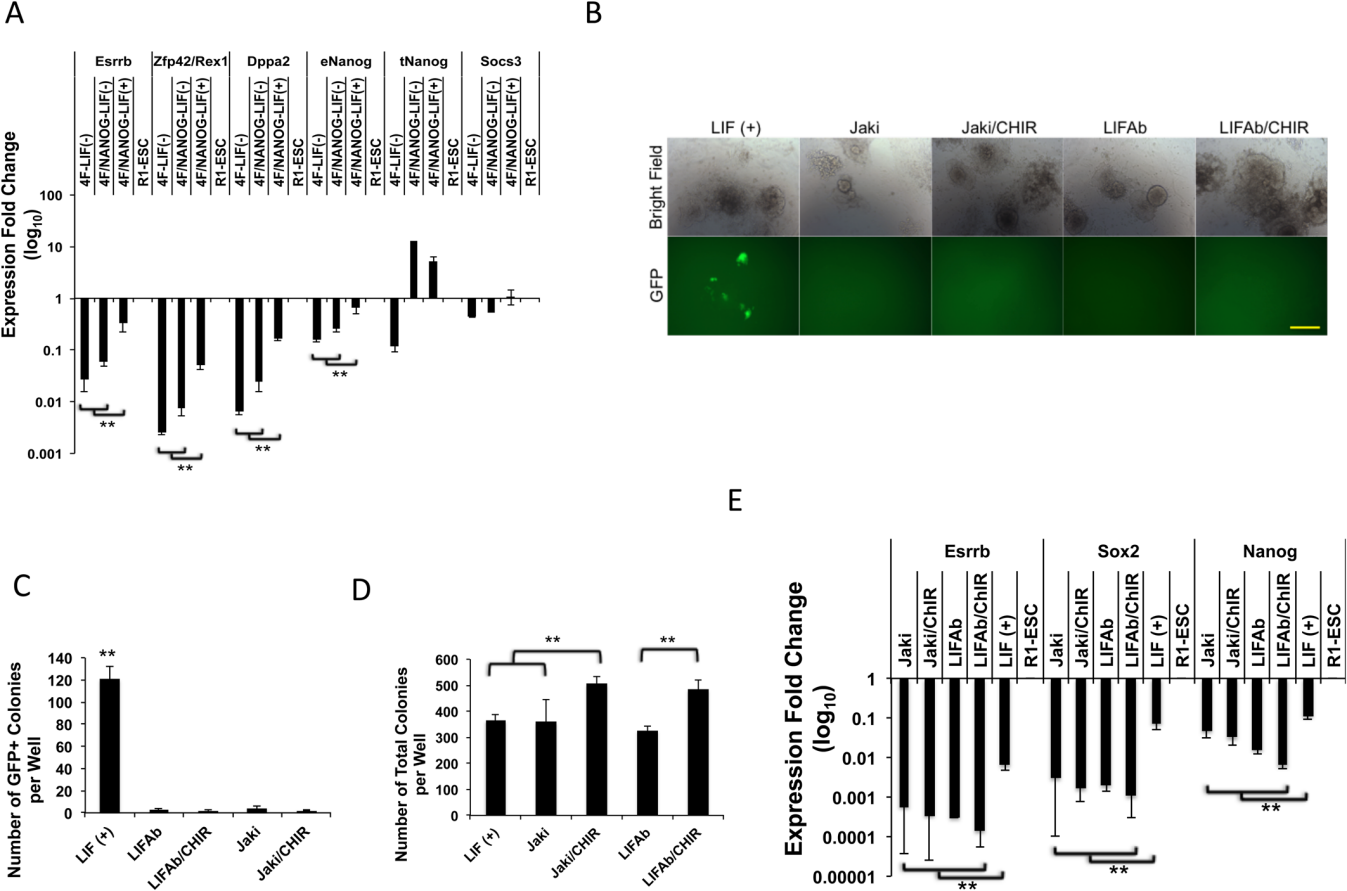
**Esrrb expression depends on LIF and Jak activity in reprogramming.** (A) qRT-PCR analysis of pluripotent genes in reprogrammed cells transduced with retroviral 4F (OKSM) or 4F plus Nanog, with or without LIF cytokine at a 3-week time point. Values are relative to R1-ESC standard. Endogenous and total (endo- plus viral expression) Nanog (eNanog and tNanog, respectively) expression are also shown. Data are mean±s.d. from two independent biological repeats. **P<0.01. (B) Representative images of pre-iPSCs seeded into 24-well plates and treated with LIF, LIF plus Jaki, LIF plus Jaki/CHIR, LIFAb or LIFAb/CHIR at day 12 of reprogramming. Scale bar: 250 μm. (C) GFP+ colonies induced from pre-iPSCs treated as described in B were counted at day 12. Data are mean±s.d. from three independent experiments. **P<0.01. (D) Number of total colonies developed from pre-iPSC reprogramming as described in B at day 12. Data are mean±s.d. from three independent experiments. **P<0.01. (E) qRT-PCR analysis for Esrrb, Sox2, and Nanog expression in pre-iPSCs treated with Jaki, Jaki plus CHIR, LIFAb or LIFAb plus CHIR at reprogramming day 12. Values are relative to R1-ESC standard. Data are mean±s.d. from three independent experiments. **P<0.01.

*Esrrb* has been reported to be regulated by *Nanog* in ESCs (Festuccia et al., 2012). In this study, we found that *Esrrb*, but not Nanog, overexpression could resume the reprogramming of pre-iPSCs with inhibited Jak/Stat3 activity (Fig. 2A; Fig. S3). This result is also consistent with our previous study showing that with the absence of LIF, the addition of *Nanog* overexpression cannot rescue the GFP+ iPSC generation from OG-MEFs transduced with retroviral OKSM (Tang et al., 2012). We then wondered whether *Nanog* would stimulate *Esrrb* expression during the reprogramming in depleted LIF signaling. qRT-PCR analysis to these previously reprogrammed samples at a 3-week time point further revealed that without LIF cytokine, there is no significant increase in *Esrrb* expression in the reprogrammed cells compared with the OKSM transduction, despite a high level of *Nanog* transgene overexpression (Fig. 3A).

Esrrb is also the Wnt pathway downstream effector that supports ESC self-renewal, which can be activated through suppression of GSK3 by a specific inhibitor CHIR99021 (CHIR) (Martello et al., 2012). We wondered whether *Esrrb* could be similarly activated during the reprogramming process without LIF pathway signaling. We added CHIR to the pre-iPSC medium treated by Jaki or LIFAb. The pre-iPSCs cultured in LIF cytokine-containing medium developed GFP+ colonies in 12 days, while the cells treated with Jaki or LIFAb remained GFP negative (Fig. 3B). The addition of CHIR to either Jaki or LIFAb condition showed no improvement on GFP+ colony generation from pre-iPSCs (Fig. 3B,C). However, CHIR increased the number of GFP-negative colonies, resulting in a significantly greater number of total colonies developed during the pre-iPSC reprogramming process (Fig. 3D; Fig. S6). qRT-PCR analysis revealed that the addition of CHIR did not activate the expression of *Esrrb* in the pre-iPSCs treated with Jaki or LIFAb (Fig. 3E). Thus, in the absence of LIF/Jak/Stat3 signaling, Wnt activity alone cannot induce *Esrrb* expression during reprogramming, even though inhibition of GSK3 does stimulate the development of partially reprogrammed colonies. Taking together, our data strongly indicate that the expression of *Esrrb* is determined by LIF/Jak activity during the reprogramming process, and Esrrb serves as a LIF/Jak downstream effector important for the generation of completely reprogrammed iPSCs.

## DISCUSSION

The LIF-regulated Jak/Stat3 pathway is important for naïve-state pluripotency establishment across species (Weinberger et al., 2016). Although many downstream targets of Stat3 have been reported, the complete understanding of Jak/Stat3 mediated pluripotency establishment has not been achieved. Jak/Stat3 signaling has been reported to regulate pluripotency in pluripotent stem cells through a number of transcription factors such as Tfcp2l1 and Klf4 (Hall et al., 2009; Martello et al., 2013; Niwa et al., 2009; Ye et al., 2013). However, how Jak/Stat3 regulates downstream targets in reprogrammed somatic cells to achieve complete pluripotency is not well understood. We found that in mouse iPSC generation, LIF-stimulated Jak activity regulates the activation of a number of key pluripotent factors such as Esrrb. To the best of our knowledge, this is the first report demonstrating *Esrrb* as a downstream target of LIF/Jak signaling in somatic cell reprogramming. *Esrrb* is a naïve-specific pluripotency marker negatively regulated by GSK3/Tcf3 in ESCs, and overexpressing it can sustain ESC pluripotency similarly to Wnt signal activation (Martello et al., 2012). *Esrrb* is also a *Nanog* target, and overexpressing *Esrrb* promotes complete reprogramming from *Nanog*-null pre-iPSCs, and sustains LIF-independent ESC self-renewal similarly to *Nanog* (Festuccia et al., 2012). We found that inhibiting Jak/Stat3 or LIF results in the lack of *Esrrb* activity, and overexpressing *Esrrb* in pre-iPSCs resumes reprogramming despite the inhibited Jak/Stat3. However, in the case of blocked LIF or Jak/Stat3 activity, overexpression of *Nanog* or mimicking the canonic Wnt signaling by inhibiting GSK3 – the two known regulators of *Esrrb* in ESCs – could not stimulate the expression of *Esrrb*, nor could they promote complete reprogramming. Our finding highlights the multiple layers of upstream control of *Esrrb* expression, which changes between the reprogramming and pluripotency maintenance stages. Our results indicate that during the reprogramming process, the activation of *Esrrb* relies on LIF-stimulated Jak/Stat3 activity. Activated Esrrb can then serve as an important LIF downstream effector driving the cells towards complete reprogramming, and becomes the essential component parallel to LIF signaling for pluripotency maintenance as previously described (Martello et al., 2012).

We also noticed that in the absence of LIF signaling, CHIR-mediated GSK3 inhibition results in increased GFP-negative colony formation in pre-iPSC reprogramming. Multiple mechanisms could be responsible for this phenomenon, as Wnt regulates many downstream targets via suppressing GSK3 activity (Beurel et al., 2015; Sokol, 2011). Firstly, relieving the GSK3 inhibition of glycogen synthase (Embi et al., 1980) may modulate glucose homeostasis and energy metabolism of reprogrammed cells in favor of fast cell proliferation. Also, GSK3 can interact with and be activated by p53 during cellular DNA damage, resulting in increased apoptotic response (Watcharasit et al., 2002). We recently showed that knockdown of *Akt3* in ESCs activates p53 signaling, leading to apoptosis and impaired cell proliferation (Wang et al., 2017a). We also found that inhibiting GSK3 promotes the reprogramming of MEFs inhibited by blocking Akt/PKB activity, which leads to cell apoptosis (Tang et al., 2014). Thus, inhibition of GSK3 can enhance the survival of reprogrammed cells, as many of them undergo p53- and other apoptotic factor-mediated cell death (Banito et al., 2009). Thirdly, the inhibition of GSK3 by Wnt signaling also results in increased nuclear β-catenin activity that is required for ESC self-renewal (Kelly et al., 2011; Wray et al., 2011). On the other hand, in addition to Esrrb, inhibition of GSK3 may also release other factors suppressed by Tcf activity, thus enhancing cell proliferation during reprogramming. The exact mechanism for this Esrrb-independent promotion of colony development would be very interesting to investigate.

Recently, a number of studies revealed that naïve-state pluripotency can also be established in human ESCs/iPSCs (Wang and Gao, 2016; Ware, 2017). However, it was also reported that unlike the naïve-pluripotent mouse ESCs, the naïve-state human cells exhibit little *Esrrb* expression, which might account for their instability in propagation compared with their mouse counterparts (Guo et al., 2016). Understanding the Esrrb-mediated naïve pluripotency maintenance, as well as its activation during reprogramming may uncover novel routes for improvement of naïve-state human pluripotent stem cells. In light of this view, it was recently reported that Esrrb activates the oxidative phosphorylation process in reprogrammed cells, which is essential for efficient reprogramming and conversion of the primed-state pluripotency into naïve-state (Sone et al., 2017). How exactly LIF/Jak/Stat3 signaling determines *Esrrb* expression during mouse iPSC generation is currently under investigation. Nevertheless, our study demonstrates that LIF/Jak signaling dictates the activation of *Esrrb* in somatic cells during reprogramming as one of its significant downstream effectors for pluripotency establishment.

## Conclusion

We identified LIF/Jak activity-specific regulation and activation of several pluripotency-predicting genes including *Esrrb*. Functional analysis revealed that *Esrrb* overexpression rescues the reprogramming halted by the inhibited LIF/Jak/Stat3 activity, and leads to the generation of pluripotent iPSCs. We further show that during the reprogramming process, *Esrrb* serves as a LIF activity-dependent downstream effector, with its expression unstimulated by Nanog or Wnt activity when LIF/Jak signaling is missing. Our data provide new insight for LIF signaling pathway-mediated pluripotency establishment in reprogramming, which are valuable for further improving the generation of naïve-state iPSCs across species.

## MATERIALS AND METHODS

### Chemicals and DNA constructs

Doxcyclin (Dox) and Jak inhibitor (Jaki) were purchased from Merck Millipore (Billierica, MA, USA). CHIR99021 and PD0325901 were purchased from SelleckChem (Houston, TX, USA). The LIF neutralizing antibody (LIFAb) was from R&D Systems. The retro- and lenti-viral vectors including pMXs-Nanog, and FUW- M2rtTA, and the viral packaging plasmids PUMVC, psPAX2 and pCMV-VSV-G (Stewart et al., 1992) were all obtained from Addgene (Cambridge, MA, USA). FUW-TetO-Esrrb and pMXs-Stat3C were described previously (Tang et al., 2012, 2014). Nr5a2 cDNA was PCR amplified using primers (forward primer: 5′-AGTTAATTAAGGATCCATGTCTTCTAATTCAGATACTGGGG-3′ and reverse primer: 5′-ACTGTGCTGGCGGCCGCTTATGCTCTTTTGGCATGCAAC-3′) and cloned into linearized pMXs vectors (Cell Biolabs, San Diego, CA, USA) using the In-Fusion kit (Clontech Inc., Mountain View, CA, USA). Lenti- and retro-viruses were prepared with 293T cells according to the protocol from Addgene and filtered with 0.8 μm filters.

### Cell culture and pre-iPSC reprogramming assay

R1-ESCs were cultured in 2i/LIF medium (Ying et al., 2008) containing N2B27 medium with 1 μM PD0325901, 3 μM CHIR99021, 1×β-mercaptoethanol (Millipore), 1000 U/ml mouse LIF (Millipore) and 0.5×penicillin/streptomycin (Invitrogen). The induced iPSCs were initially cultured in knockout serum replacement (KSR)-ESC medium after picking and switched to 2i/LIF medium from passage 2. The KSR-ESC medium consists of 76% knockout-DMEM, 20% KSR, 1% 100×Glutamax, 1% 100×non-essential amino acids, 0.5×penicillin/streptomycin (all from Invitrogen) and supplemented with 1% 100×β-mercaptoethanol and 1000 U/ml mouse LIF.

Generation of the Jaki-treated pre-iPSCs was described previously (Tang et al., 2012), where the OG-MEFs were reprogrammed with OKSM in the presence of 1 μM Jaki. Single pre-iPSC colonies were picked and expanded in KSR-ESC medium containing mouse LIF and 1 μM Jaki (thereafter called KSR-ESC-Jaki medium). Reprogramming assay was performed in KSR-ESC-Jaki medium or the KSR-ESC medium containing no LIF but 2.5 μg/ml mouse LIF neutralizing antibodies (KSR-ESC-LIFAb medium). For the reprogramming assay, on day 1, 0.25 million pre-iPSCs were seeded into a 24-well plate in which mitomycin C-treated CD1 MEF feeders were plated beforehand. On day 0, the cells were infected with retro- or lenti-viral vector control or the genes of interest in the presence of polybrene (American BIO, Natick, MA, USA) overnight. Starting from day 1, KSR-ESC-Jaki medium or the KSR-ESC-LIFAb medium was applied for reprogramming. Application of CHIR99021 for WNT activation or Dox for induced expression was started on day 2. Media were replaced every other day. GFP-expressing colonies were counted between 12 days to 3 weeks after initial viral transduction under a Nikon fluorescence microscope. GFP-positive iPSC colonies were picked at 3 weeks after viral transduction and expanded for further characterization.

### EB formation

Established iPSCs lines (passage 3) were passaged onto CD1 MEF feeders. Colonies were trypsinized and single cells were plated back to the tissue culture dish for 2 h to allow MEFs to attach. The iPSC cells in supernatant were then transferred to a low adhesive Petri-dish and allowed to form EBs and differentiate in 10% FBS in DMEM without LIF. Upon 1 week of differentiation, the EBs were re-plated to 0.1% gelatin (Millipore)-coated tissue culture dish for another week before proceeding to RNA extraction and qRT-PCR.

### qRT-PCR analysis

Total RNAs were extracted using Trizol (Invitrogen), and 1 μg of the total RNAs was reverse transcribed with All-in-One cDNA Synthesis SuperMix (Bimake, Houston, TX, USA). For qRT-PCR, 2× SYBR Green PCR Master Mix (Bimake) was used. Samples were run using an ABI 7500 Fast instrument, and data were analyzed using the 7500 software (version 2.0.2) provided with the instrument. All genes were normalized with GAPDH as internal control and relative mRNA expression was quantified using R1-ESCs as the reference as specified in each figure legend.

### Immunostaining

The mouse iPSCs differentiated with the EB-mediated method in Gelatin-coated dishes were fixed with 4% paraformaldehyde plus 1% sucrose in PBS, after which the cells were treated with 0.5% TX-100 to permeabilize the cell membrane and blocked with donkey serum. Then, the cells were incubated with the antibodies (R&D System) against three germ layer makers including Otx2 for ectoderm, Brachyury for mesoderm and Gata6 for endoderm. The cell nuclei were counterstained with DAPI and fluorescent images were visualized using a Nikon fluorescent microscope.

### Data analysis

The RNA-seq data were from a previous study (GEO accession number GSE97261) (Wang et al., 2017b). Data analyzed through Pearson correlation coefficient were created by R Package, which was in turn used to generate the heatmap. qRT-PCR and cell counting data were processed using one-Way ANOVA with Tukey's multiple comparisons or the Student's *t*-test. Data are presented as mean±standard deviation (s.d.). *P*<0.05 was considered statistically significant.

## Supplementary Material

Supplementary information
