# Supplementary material for: LIF-activated Jak signaling determines Esrrb expression during late-stage reprogramming
Source: Biol Open. 2017 Dec 6;7(1):bio029264. doi: 10.1242/bio.029264 (PMC5829498; doi:10.1242/bio.029264)
Supplement: Supplementary information [file biolopen-7-029264-s1.pdf]

Supplementary Figures

Fig. S1: Schematic diagram depicting the MEF reprogramming process and dates for LIF and LIFAb treatments and sample collection.

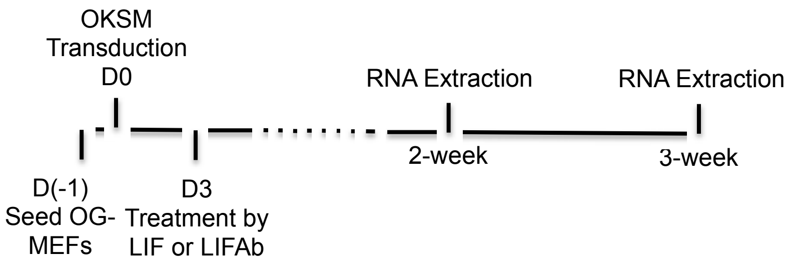

Fig. S2: Key Pluripotent Genes Downregulated by Blocking LIF Signaling in Reprogramming.

qRT-PCR analysis of key pluripotent gene expressions in MEF reprogramming treated with LIF or LIFAb at 2- and 3-week time points. Bars represent mean from triplicate wells. R1-ESC was used as the control.

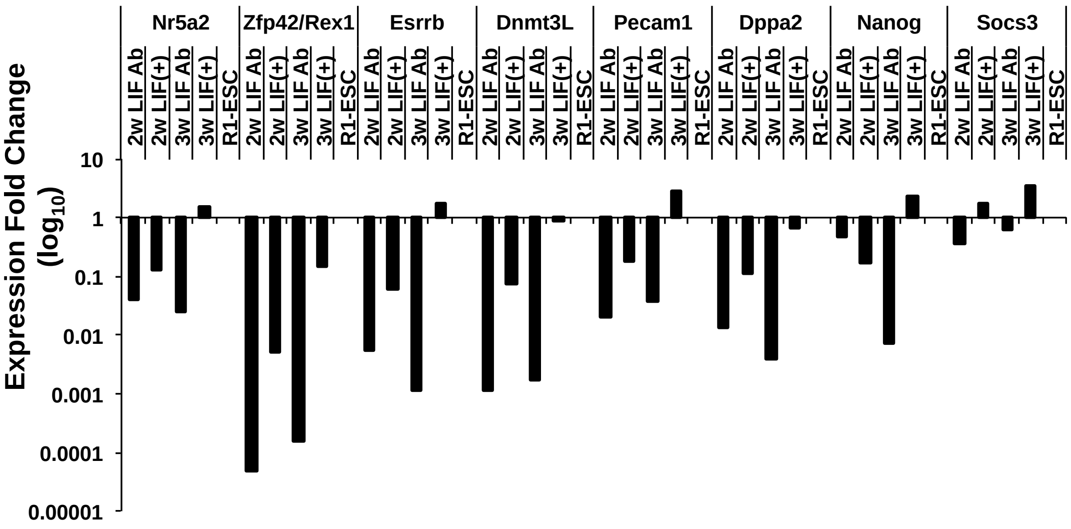

Biology Open • Supplementary information

Fig. S3: Esrrb Promotes Complete Reprogramming Despite Jak/Stat3 Inhibition.

Jaki-treated pre-iPSC lines (P6J1 and P6J2) were expanded and seeded into 24-well-plates and infected with vector Ctl or virally expressed Esrrb, Nanog, Klf2, Nr5a2, Lin28, or Prdm14 and cultured in the presence of LIF or LIF plus Jaki. GFP+ colonies were counted at 2 weeks after viral infection. Bars represent mean±SD from two independent experiments. **: p < 0.01.

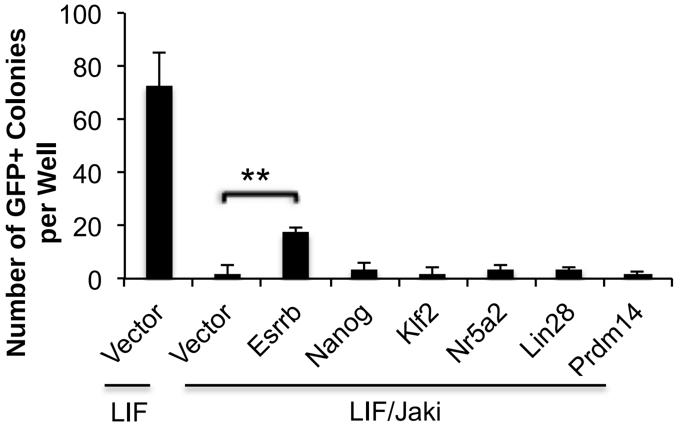

Fig. S4: Putative Esrrb-induced iPSCs Express Pluripotent Markers.

qRT-PCR analysis of endogenous pluripotent markers for Esrrb induced putative iPSC colonies and the parental pre-iPSCs. R1-ESC was used as the Ctl. Bars represent mean±SD from two different cell lines derived.

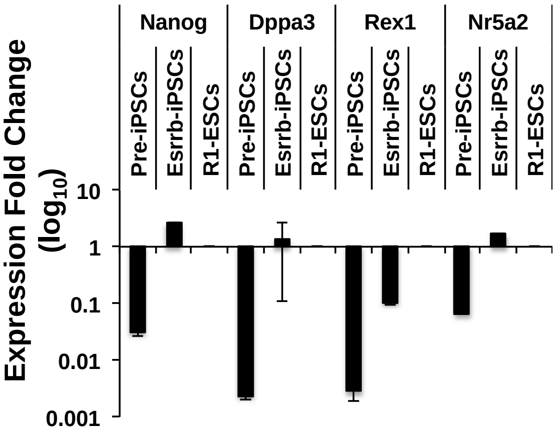

Biology Open • Supplementary information

Fig. S5: Three-Germ Layer Differentiation of Esrrb-induced Putative iPSCs (Immunostaining).

Immunostaining for the expression of the three germ layer markers at day 14 of EB differentiation (Endoderm: Gata4; Mesoderm: Brachyury; Ectoderm: Otx2). Primary antibodies were conjugated with NL493 or NL557 fluorochromes. Cell nuclei were counterstained with DAPI. The differentiated cells not stained with antibodies (Ctl) showed no NL493 and NL557 fluorescence signals. (Bar = 120 μm).

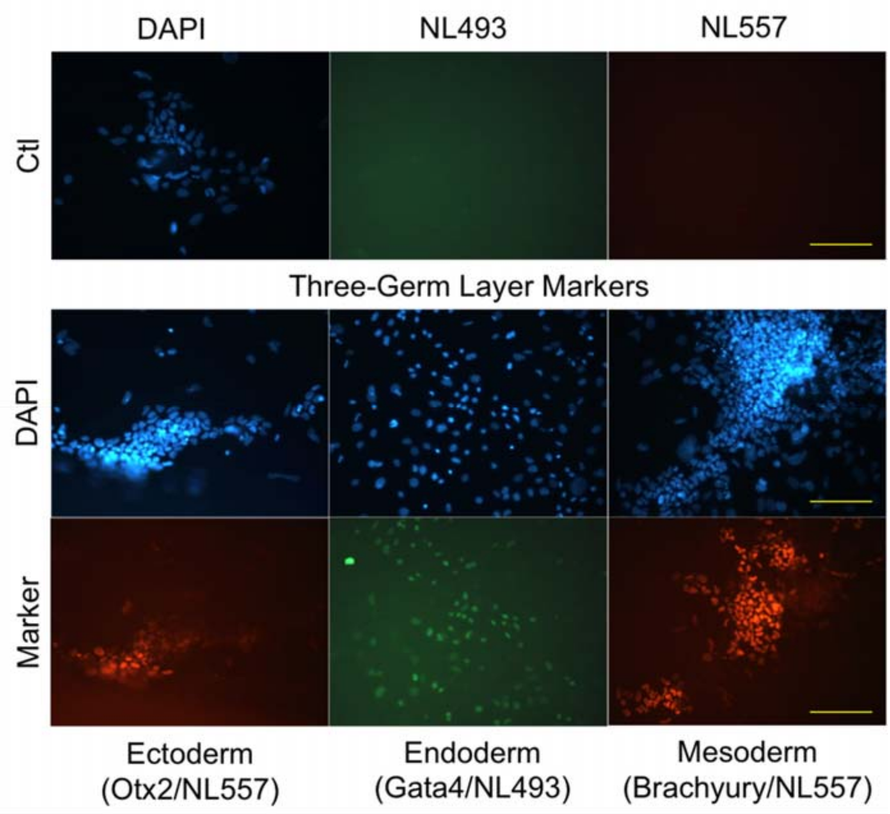

Biology Open • Supplementary information

Fig. S6: Representative images of pre-iPSCs seeded into 24-well-plates and treated with LIF plus Jaki, LIF plus Jaki/CHIR, LIFAb, or LIFAb/CHIR at day 12 (Bar = 625 µm).

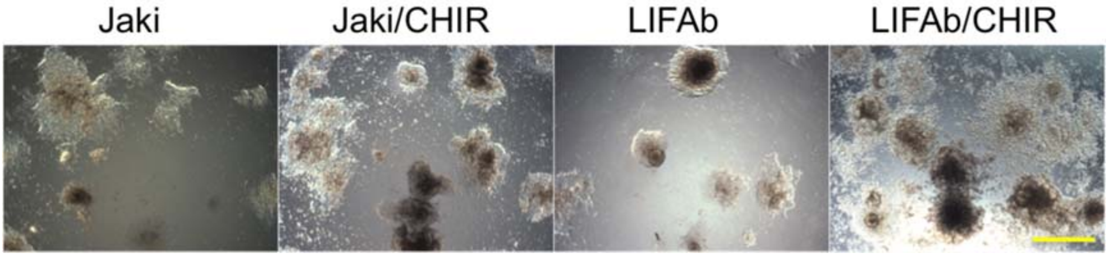

Supplementary Online Video 1

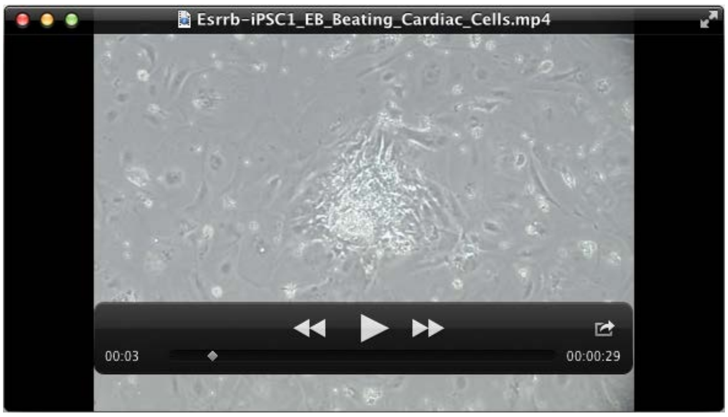

Supplementary online Video 1: Beating cardiac cell formation from Esrrb-induced iPSCs upon EB formation at day 14.
